# Dissecting grain yield pathways and their interactions with grain dry matter content by a two-step correlation approach with maize seedling transcriptome

**DOI:** 10.1186/1471-2229-10-63

**Published:** 2010-04-12

**Authors:** Junjie Fu, Alexander Thiemann, Tobias A Schrag, Albrecht E Melchinger, Stefan Scholten, Matthias Frisch

**Affiliations:** 1 Institute of Plant Breeding, Seed Science and Population Genetics, University of Hohenheim, 70599 Stuttgart, Germany; 2 Biocenter Klein Flottbek, University of Hamburg, 22609 Hamburg, Germany; 3 Institute of Agronomy and Plant Breeding II, Justus Liebig University, 35392 Giessen, Germany

## Abstract

**Background:**

The importance of maize for human and animal nutrition, but also as a source for bio-energy is rapidly increasing. Maize yield is a quantitative trait controlled by many genes with small effects, spread throughout the genome. The precise location of the genes and the identity of the gene networks underlying maize grain yield is unknown. The objective of our study was to contribute to the knowledge of these genes and gene networks by transcription profiling with microarrays.

**Results:**

We assessed the grain yield and grain dry matter content (an indicator for early maturity) of 98 maize hybrids in multi-environment field trials. The gene expression in seedlings of the parental inbred lines, which have four different genetic backgrounds, was assessed with genome-scale oligonucleotide arrays. We identified genes associated with grain yield and grain dry matter content using a newly developed two-step correlation approach and found overlapping gene networks for both traits. The underlying metabolic pathways and biological processes were elucidated. Genes involved in sucrose degradation and glycolysis, as well as genes involved in cell expansion and endocycle were found to be associated with grain yield.

**Conclusions:**

Our results indicate that the capability of providing energy and substrates, as well as expanding the cell at the seedling stage, highly influences the grain yield of hybrids. Knowledge of these genes underlying grain yield in maize can contribute to the development of new high yielding varieties.

## Background

Maize production in 2007 was about 800 million tonnes - more than rice or wheat http://faostat.fao.org, and it is likely to become the most important source for human nutrition by 2020 [1]. Conventional breeding approaches employing direct phenotypic selection with limited or no knowledge of the underlying morpho-physiological determinants have successfully improved yield [2]. Maize grain yield and its major components - kernel weight, kernel number per ear, ear number per plant - have been studied by quantitative trait locus (QTL) mapping approaches [3]. The identified chromosome regions provide a starting point for further decoding the mechanisms affecting maize production. In European maize breeding, early maturity of high yielding varieties is an important breeding goal, since the short growing season limits productivity. Therefore, grain dry matter content, as an indicator for early maturity, is a major factor determining maize productivity.

Genes directly involved in grain yield, including those associated with grain number (e.g., *OsCKX2*), grain weight (e.g., *GS3 *and *GW2*) and grain filling were identified in rice ([4] for review). Further, genes indirectly associated with grain yield via plant height (e.g., *Rht1*, *sd1*, and *BRI1*) and tillering (e.g., *TB1*, *FC1*, and *MOC1*) were also identified. These findings underline the important roles of cell cycle, phytohormone signaling, carbohydrate supply, and the ubiquitin pathway and have increased our understanding of grain yield. However, the mechanisms and pathways controlling yield and yield-related traits still remain largely unknown.

Genome-scale oligonucleotide arrays have become a powerful tool in detecting the pathways and pathway interactions underlying biological processes. In maize, results on ear and kernel development have been reported [5,6]. However, no results focusing on maize yield or early maturity are available.

Our objectives were to investigate the genes and gene networks underlying grain yield in maize, and their interaction with genes underlying grain dry matter content, by employing a newly developed two-step correlation analysis that combines multi-environment field data and transcription profiles.

## Results

### Grain yield-involved genes

The modified *F*-test with a false discovery rate (FDR) of 0.01 [7] revealed that 12,288 out of the 43,381 gene-oriented probes representing complementary maize genes were differentially expressed in the parental inbred lines of the 98 hybrids. For 10,810 among them, the fold change was greater 1.3 and the log-2 expression intensity was greater 8.0. This set of significant differentially expressed genes was subjected to further analyses. The average number of genes differentially expressed between the parents of a hybrid was 3350, which equals 7.7% of the genes on the array (see Additional file 1).

**Figure 1:**
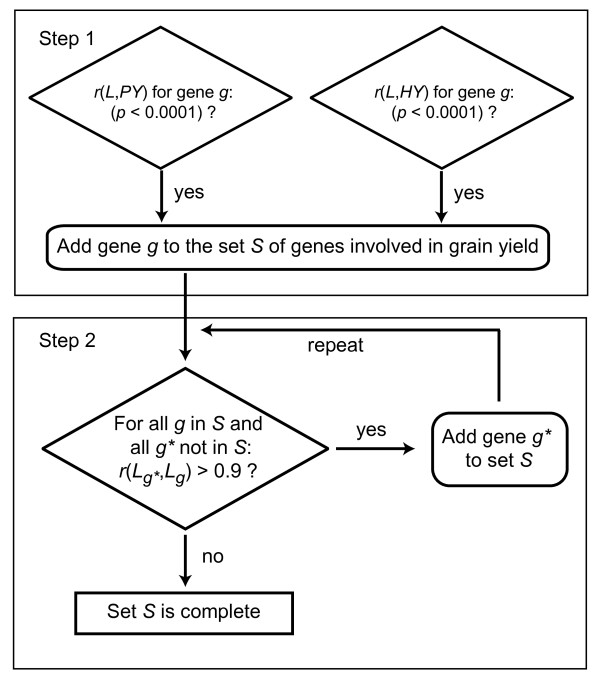
**Schematic representation of a two-step correlation approach**. *L*, average expression level of a gene in the parents of a hybrid; g*, gene not included in set *S *in a previous repetition of Step 2; *r*, correlation coefficient; *p*, *p*-value for statistical significance; *PY*, hybrid performance for grain yield; *HY*, mid-parent heterosis for grain yield.

The mid-parent expression level of 2511 differentially expressed genes was significantly (*p *< 0.01) correlated with hybrid performance (*PY*) or heterosis (*HY*) for grain yield. In Step 1 of the two-step selection approach (Figure 1), 540 genes were found to be highly significantly (*p *< 0.0001) correlated with *PY *or *HY*. In Step 2, additional 205 genes were added to the set of grain yield associated genes *S*. The gene expression of 468 genes (62.8% of 745 genes) was positively and that of 277 (37%) negatively correlated with *PY *(see Additional file 2). Note however, that these percentages are based on probes and may overestimate the actual number of differentially regulated genes, because there may not always be a one-to-one relationship between probes and genes.

With information from the Swissprot Knowledgebase, we found that 18 of the grain yield associated genes were identical to known maize genes, including *IVR1 *encoding invertase (MZ00005490), *GLU1 *(MZ00035426), *PHI1 *(MZ00014260), *RBCS *(MZ00014822), and *HDT3 *encoding histone deacetylase (MZ00023941). Furthermore, a high correlation (*r *> 0.6) was observed for genes encoding hexokinase (MZ00042300) and phosphofructokinase/PFK (MZ00013816), a dynamin-related gene (MZ00014057), and MZ00026127 (*OsNAC4 *homologue) well-known as a transcription factor gene involved in the regulation of developmental processes [8].

In a cross validation procedure, three of the seven flint lines and five of the fourteen dent lines were randomly sampled with 100 repetitions. On average 190 of the 200 genes showing the strongest correlation with *PY *in the estimation set were among the set of the 200 genes with the strongest correlation in the complete data set. For *HY *the average number of agreeing genes was 185. This result confirms that the different genetic backgrounds of the inbred lines only marginally contributed to the random error in the correlation analysis.

### Interaction between grain yield and grain dry matter content associated genes

The negative correlation *r*(*PY*, *PD*) = -0.410 between hybrid performance for grain yield and grain dry matter content was significant (*p *= 0.002). This suggests that the gene networks involved in grain yield and grain dry matter content might be overlapping and negatively interacting with each other. Employing the two-step selection approach (Figure 1) we detected 622 genes associated with grain dry matter content. A total of 103 genes had an influence on both traits and had correlations of opposite sign with regard to grain dry matter content and grain yield (see Additional file 2). Some of these genes were located in the phytohormone signaling pathways (e.g., auxin-responsive factor, beta-glucosidase) and the flavonoid metabolism (e.g., isoflavone reductase, 2-hydroxyisoflavanone dehydratase; Table 1).

**Table 1:** The list of selected genes involved in grain yield.

Probe ID	Annotation	Mean	FD	Association		Step	Ref^§^	Figure
				grain yield	GDMC			
MZ00013618	CIPK9-like protein	9.0	1.7	P	-	F	[3]	
MZ00014057	Dynamin-related protein 1A, putative	9.8	2.0	P	N	F		Fig. 3
MZ00014612	ARID/BRIGHT DNA-binding domain-containing protein, putative	7.8	1.6	N	P	F		
MZ00014822	Ribulosebisphosphate carboxylase. {Zea mays;}	9.7	3.7	N	-	F		
MZ00015132	O-methyltransferase ZRP4 (EC 2.1.1.-) (OMT) {Zea mays}	8.9	2.1	P	-	F	[1]	
MZ00016342	SEUSS transcriptional co-regulator, homologue	9.3	1.4	N	P	F		
MZ00017365	Serine/threonine-protein kinase SAPK3, putative	10.3	1.5	P	-	F		
MZ00018334	High light protein {Hordeum vulgare}	8.8	1.5	P	-	F		
MZ00018444	2-Hydroxyisoflavanone dehydratase, putative	8.4	1.6	P	N	F		
MZ00018517	2-Hydroxyisoflavanone dehydratase, putative	10.7	2.8	P	N	F		
MZ00020198	Thioredoxin M-type, chloroplast precursor (TRX-M) {Zea mays}	13.3	2.1	N	-	S		
MZ00021090	DNA-3-methyladenine glycosylase (MAG), homologue	8.4	1.4	P	-	F		
MZ00022903	Leucine-rich repeat transmembrane protein kinase, putative	8.6	2.7	N	-	F		
MZ00023941	Histone deacetylase 2c (Zm-HD2c) {Zea mays}	8.2	3.4	P	-	S		
MZ00024407	Agamous-like MADS box protein AGL9 homolog, putative	7.5	1.4	P	N	F		
MZ00026127	Development regulation gene OsNAC4, homologue	9.2	1.8	P	-	F		
MZ00026879	Putative receptor-mediated endocytosis 1 isoform I/calcium-binding EF hand family protein	10.5	1.3	N	-	F		
MZ00029320	Isoflavone reductase homolog, putative	9.5	6.2	P	N	S		
MZ00033058	Plasma membrane ATPase 1, putative	8.2	1.7	N	-	F		
MZ00044236	Putative calcium-dependent protein kinase	8.9	1.5	P	-	F		
MZ00046983	Glycosyl transferase family 17 protein, putative	8.3	1.3	N	-	F		
MZ00056596	24-methylenesterol C-methyltransferase 2(SMT2), homologue	8.8	2.1	N	-	F		Fig. 3
MZ00057130	Dof-type zinc finger domain-containing/OBP1-like protein, orthologue	8.0	1.9	P	-	F		
MZ00057320	Putative ribulose-5-phosphate-3-epimerase	9.0	1.6	P	-	F	[3]	
**Carbohydrates and energy**								
MZ00005490	Beta-fructofuranosidase/vacuolar invertase {Zea mays}	8.2	1.9	P	-	F	[1]	Fig. 2
MZ00013514	UDP-glucose pyrophosphorylase, homolgue	8.2	1.5	P	-	F		Fig. 2
MZ00013816	Adenosine kinase/phosphofructokinase (PFK) {Zea mays}	9.9	3.3	P	-	F		Fig. 2
MZ00014260	Glucose-6-phosphate isomerase, cytosolic {Zea mays}	11.2	1.6	N	-	F		Fig. 2
MZ00015645	Pyrophosphate-fructose 6-phosphate 1-phosphotransferase (PFP) alpha subunit, putative	8.7	1.6	N	-	F	[1]	Fig. 2
MZ00017454	Putative GDP-mannose pyrophosphorylase	10.1	1.5	N	-	F		Fig. 2
MZ00024012	Pyrophosphate-fructose 6-phosphate 1-phosphotransferase (PFP) beta subunit, putative	10.7	2.7	P	-	F	[3]	Fig. 2
MZ00024213	Pyrophosphate-fructose 6-phosphate 1-phosphotransferase (PFP) alpha subunit, putative	12.0	1.6	P	-	F		Fig. 2
MZ00026683	Putative beta-fructofuranosidase/cytosolic invertase	10.3	1.4	P	-	F		Fig. 2
MZ00033179	Beta-fructofuranosidase/cell wall invertase {Zea mays}	8.8	2.0	P	-	F	[2]	Fig. 2
MZ00036953	Triosephosphate isomerase, cytosolic, putative	9.7	3.1	N	P	S	[3]	Fig. 2
MZ00039244	Phosphoglycerate kinase, putative	10.4	1.7	P	-	F		Fig. 2
MZ00042300	Putative hexokinase (HXK)	8.9	1.4	P	N	F		Fig. 2
**Cell cycle, DNA processing, and cell fate**								
MZ00004156	Endo-1,3-beta-D-glucosidase, putative	9.0	1.9	P	-	F		Fig. 3
MZ00013343	Histone H4, similarity	12.3	1.8	P	-	F	[2,3]	Fig. 3
MZ00013961	V-type H+ATPase, putative	7.7	1.4	P	-	F		Fig. 3
MZ00017273	CDK regulatory subunit	9.2	2.1	P	-	S		
MZ00017440	CDC2/B-type CDK, homologue	8.5	2.9	N	-	S		Fig. 3
MZ00017840	DNA ligase, putative	9.0	1.6	P	-	F		Fig. 3
MZ00017975	CDK-activating kinase assembly factor-related	9.2	1.3	P	N	F		
MZ00021340	Putative beta-expansin	8.1	1.4	P	-	F	[2]	Fig. 3
MZ00021442	Cyclin-dependent kinase inhibitor 7 (ICK7), homologue	8.9	1.5	P	-	F		Fig. 3
MZ00022872	Putative beta-expansin	8.3	1.7	P	-	F	[3]	Fig. 3
MZ00026530	Enhancer of rudimentary, putative	9.7	3.0	P	-	F		Fig. 3
MZ00027266	Putative cell division protein FtsZ (CH)	10.1	1.5	P	-	S		Fig. 3
MZ00027598	Putative replication factor subunit	9.5	1.8	P	-	F		Fig. 3
MZ00030457	Putative alpha-expansin	8.3	1.3	P	-	F		Fig. 3
MZ00030567	Putative alpha-expansin 1 precursor	8.5	2.1	N	-	F	[1,3]	Fig. 3
MZ00041750	Prolifera protein (PRL)/DNA replication licensing factor Mcm7 (MCM7)	8.7	2.4	P	-	F	[3]	Fig. 3
MZ00043527	Aquaporins/tonoplast membrane integral protein ZmTIP3-1 {Zea mays}	8.2	2.8	P	-	F	[3]	Fig. 3
MZ00044246	Putative CDC48-like protein	8.6	1.5	P	-	F		
**Ubiquitin pathway**								
MZ00000787	F-box/tubby family protein, putative	8.7	2.0	P	-	F	[1]	Fig. 3
MZ00012603	RWD domain containing 1-like protein, putative	8.9	1.8	P	N	F		
MZ00012765	RING finger subunit, putative	7.6	1.9	P	N	F		Fig. 3
MZ00020431	E3 ubiquitin ligase APC1, putative	8.1	1.5	P	-	F		Fig. 3
MZ00026276	Ubiquitin-conjugating enzyme E2-17 kDa, putative	9.2	2.4	P	N	S	[3]	
MZ00030283*	CCS52A class, homologue	8.5	1.2	P	-			Fig. 3
MZ00036978	SKP1 family, putative	11.0	1.9	N	-	F		
MZ00039271	F-box/LRR protein, putative	8.9	1.6	P	-	F		Fig. 3
MZ00056403	Ubiquitin-conjugating enzyme E2-17 kDa, putative	9.7	2.0	P	-	S	[3]	
**Phytohormone pathway**								
MZ00003819	Putative ethylene-responsive transcriptional coactivator (MBF1)	8.7	2.7	P	-	F	[1]	Fig. 3
MZ00012636	Glutathione S-transferase GST 29 (auxin-induced) {Zea mays}	8.4	2.0	N	-	F		
MZ00013540	14-3-3-like protein, putative	10.5	3.1	P	-	F		
MZ00013608	Beta-glucosidase aggregating factor {Zea mays}	12.1	2.8	P	-	F	[2,3]	
MZ00014891	Contains similarity to gibberellin-stimulated transcript 1 like protein, putative	8.7	1.5	P	-	F	[3]	
MZ00018299	Ethylene-responsive protein, putative	8.7	1.5	P	-	F		
MZ00021497	Auxin-responsive family protein, putative	8.7	1.3	P	-	F		
MZ00024781	Putative auxin-responsive factor (ARF1)	8.5	1.4	P	-	S	[2]	
MZ00025819	BRI1-associated receptor, homologue	10.0	1.8	P	-	F		
MZ00026772	bHLH/IAA-LEUCINE RESISTANT3, homologue	10.4	1.5	N	P	S		
MZ00028517	Abscisic acid-insensitive 4 (ABI4)-like protein, putative	7.6	1.3	P	-	F		
MZ00030444	Glutathione S-transferase, putative	9.1	1.3	P	N	F		
MZ00031351	Two-component responsive regulator 2/response regulator 4 (ARR4)-like protein {Zea mays}	9.4	1.7	P	-	F		
MZ00034947	Glycosyl hydrolase family 1/Beta-glucosidase-like protein, putative	8.6	1.2	N	P	F		
MZ00035426	Beta-glucosidase {Zea mays}	8.0	2.6	P	N	F	[2]	
MZ00038300	Auxin response factor 2, putative	7.9	3.2	P	-	S		
MZ00040986	IAA-alanine resistance protein, putative	8.1	1.2	N	-	F		
MZ00044325	Auxin-responsive protein -related, similarity	10.4	2.2	P	N	S		
**Stress**								
MZ00001535	Heat shock protein, putative	8.0	1.6	N	P	F		
MZ00004615	Pathogenesis-related protein, putative	10.1	3.3	P	-	F		
MZ00013860	DNAJ heat shock protein, putative	10.5	2.3	P	-	F		
MZ00017699	Putative drought-induced protein, related	10.5	2.0	P	-	F		
MZ00022225	AN1-like protein/ZmAN18 {Zea mays}	9.9	3.1	P	-	F		
MZ00036400	LEA3 family protein, putative	10.6	2.2	P	N	F		
MZ00056817	Cold-shock DNA-binding family protein, homologue	8.2	1.6	P	-	F		
**Transporter**								
MZ00017748	Putative peptide transporter	12.2	1.7	P	-	F		
MZ00018481	Putative Potassium channel protein	9.3	1.7	P	-	F	[1]	
MZ00026499	Glucose-6-phosphate/phosphate-translocator precursor, homolog	10.0	1.6	P	-	F		
MZ00043904	ABC transporter family protein	9.0	1.8	P	-	F		

The grain yield-involved genes are collected in Step 1 (F) and Step 2 (S). For each gene, mean and fold-change (FD) of mid-parent expression are calculated; the positive (P) and negative (N) association to grain yield and grain dry matter content (GDMC) are also provided.

§1, Fernandes et al., 2008 [10]; 2, Zhu et al., 2009 [6]; 3, Liu et al., 2008 [5].

* Probes (genes) with marginal significance included for discussion.

Among the interacting genes, only 39 genes were identified in Step 1. However, 64 more genes were included in Step 2. About half of these additional genes were associated with only one trait (grain yield or grain dry matter content) at the 0.0001 level, but were highly correlated with a significant gene concerning the second trait.

### Functional classification of trait-involved genes

To examine the functions of grain yield and grain dry matter content associated genes, these were grouped into functional categories based on the MIPS Functional Catalogue (Table 2, Additional file 2). The functional category METABOLISM contained most of the genes for both traits. For grain yield, it was followed by PROTEIN WITH BINDING FUNCTION OR COFACTOR REQUIREMENT and for grain dry matter content by CELL RESCUE, DEFENSE AND VIRULENCE. Furthermore a large number of genes were related to processes involved in ENERGY. In Step 2 of the selection approach, the additional genes in categories CELL CYCLE AND DNA PROCESSING and CELL FATE were included in the set of grain yield associated genes, resulting in an enrichment of these two categories. The category CELL RESCUE, DEFENSE AND VIRULENCE included the largest number of genes, which were associated with both traits.

**Table 2:** The distribution of trait-involved genes in the MIPS Functional Catalogue.

Functional category	Background (DG)		grain yield- involved				GDMC-interacted		GDMC-involved	
				
			Step 1		Step 2		Step 2		Step 2	
	n	%	n	%	n	%	n	%	n	%
METABOLISM	858	*31.1%*	39	*29.5%*	52	*29.2%*	4	*19.0%*	**54**	*37.2%*
ENERGY	281	10.2%	17	12.9%	21	11.8%	2	9.5%	**21**	14.5%
CELL CYCLE AND DNA PROCESSING	153	5.5%	7	5.3%	**14**	7.9%	2	9.5%	**13**	9.0%
										
TRANSCRIPTION	266	9.6%	14	10.6%	20	11.2%	2	9.5%	15	10.3%
PROTEIN SYNTHESIS	336	12.2%	15	11.4%	21	11.8%	1	4.8%	13	9.0%
PROTEIN FATE	324	11.7%	10	7.6%	18	10.1%	3	14.3%	18	12.4%
PROTEIN WITH BINDING FUNCTION OR COFACTOR REQUIREMENT	376	*13.6%*	22	*16.7%*	**29**	*16.3%*	1	4.8%	17	11.7%
										
CELLULAR TRANSPORT, TRANSPORT FACILITATION AND TRANSPORT ROUTES	360	13.0%	**22**	*16.7%*	27	15.2%	3	14.3%	15	10.3%
CELLULAR COMMUNICATION/SIGNAL TRANSDUCTION MECHANISM	322	11.7%	16	12.1%	20	11.2%	1	4.8%	11	7.6%
CELL RESCUE, DEFENSE AND VIRULENCE	307	11.1%	10	7.6%	14	7.9%	5	*23.8%*	**27**	*18.6%*
INTERACTION WITH THE CELLULAR ENVIRONMENT	86	3.1%	6	4.5%	6	3.4%	-	-	3	2.1%
INTERACTION WITH THE ENVIRONMENT	83	3.0%	3	2.3%	3	1.7%	-	-	3	2.1%
CELL FATE	159	5.8%	10	7.6%	**15**	8.4%	1	4.8%	8	5.5%
DEVELOPMENT	160	5.8%	9	6.8%	10	5.6%	-	-	9	6.2%
BIOGENESIS OF CELLULAR COMPONENTS	289	10.5%	11	8.3%	20	11.2%	3	14.3%	15	10.3%

DG, differentially expressed genes; grain yield-involved, genes involved in grain yield; GDMC-interaction, the grain yield-involved genes which negatively interacted with grain dry matter content; GDMC-involved, genes involved in grain dry matter content; n, number of genes; *p*, *p*-value for statistical significance. The symbol "-" represents data unavailable. The numbers in boldface represent significance at *p *< 0.05. The percentages in italics represent the first two largest categories in each set of genes.

### Significantly regulated metabolic pathways

**Figure 2:**
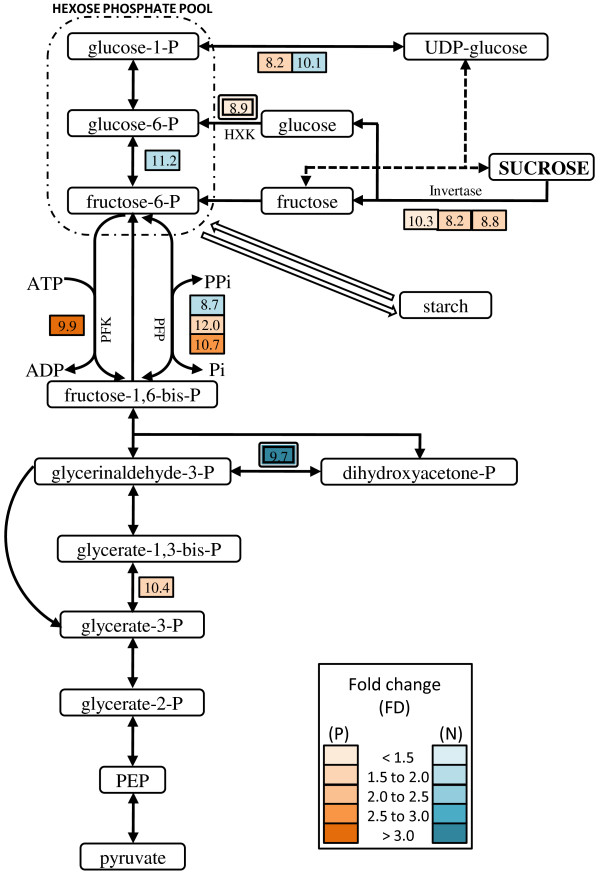
**Representation of grain yield-involved genes in sucrose degradation and glycolysis pathways**. The rectangular boxes with the colored scales show the fold-changes (FD) of mid-parent expression for each gene. The mean mid-parent expression (log2 scale) is represented by the numbers in the boxes. Positively (P) and negatively (N) associated genes are shown in brown and blue, respectively. The boxes with two frames show genes with interactions to grain dry matter content (GDMC).

In an enrichment analysis of the grain yield associated genes with RiceCyc, we determined overrepresented pathways. These included sucrose degradation, cyclopropane and cyclopropene fatty acid biosynthesis, and plant respiration (Table 3, Additional file 2). Many grain yield associated genes were classified to the pathways of glycolysis, fructose degradation to pyruvate and lactate, glucose fermentation to lactate, and the Calvin cycle. Two genes were involved in the biosynthesis of the growth hormone IAA, one of these two genes was associated with both grain yield and grain dry matter content. One gene (MZ00042300) coding for a hexokinase involved in the degradation of sugars (e.g. sucrose), was associated with both traits (Figure 2).

**Table 3:** Statistical enrichment analyses of metabolic pathways.

Metabolic pathway	Back-ground	grain yield- involved				GDMC-interacted		GDMC-involved	
				
		Step 1		Step 2		Step 2		Step 2	
	n	n	*p*	n	*p*	n	*p*	n	*p*
Acyl-CoA thioesterase pathway	7	1	2.6E-1	1	3.2E-1	**1**	4.5E-2	1	2.7E-1
Aerobic respiration -- electron donor II	36	3	1.7E-1	**7**	7.4E-3	-	-	2	2.8E-1
Aerobic respiration -- electron donor III	18	**3**	4.8E-2	**7**	9.6E-5	-	-	-	-
Betanidin degradation	70	5	1.4E-1	6	1.5E-1	1	3.1E-1	5	1.5E-1
Calvin cycle (CO2 fixation)	35	4	6.9E-2	5	6.0E-2	1	1.9E-1	2	2.8E-1
Chlorophyllide *a *biosynthesis	24	3	8.9E-2	**5**	1.7E-2	1	1.4E-1	3	9.6E-2
Cyanate degradation	13	2	1.1E-1	**3**	4.6E-2	-	-	1	3.6E-1
Cyclopropane and cyclopropene fatty acid biosynthesis	12	1	3.5E-1	**3**	3.8E-2	-	-	-	-
									
DIMBOA-glucoside degradation	3	1	1.4E-1	1	1.8E-1	**1**	2.0E-2	1	1.4E-1
Fructose degradation to pyruvate and lactate (anaerobic)	72	5	1.5E-1	6	1.6E-1	1	3.1E-1	**8**	2.1E-2
Glucose fermentation to lactate II	56	5	9.1E-2	5	1.6E-1	1	2.7E-1	**6**	4.6E-2
Glutathione redox reactions I	7	-	-	1	3.2E-1	**1**	4.5E-2	1	2.7E-1
Glycolysis I	69	6	7.7E-2	7	9.8E-2	1	3.1E-1	**7**	4.2E-2
Glycolysis IV (plant cytosol)	63	5	1.2E-1	6	1.3E-1	1	2.9E-1	**7**	2.9E-2
IAA biosynthesis VI (via indole-3-acetamide)	6	-	-	2	5.4E-2	**1**	3.9E-2	1	2.4E-1
									
Mannose degradation	1	1	5.1E-2	1	7.0E-2	**1**	6.7E-3	1	5.3E-2
Btarch degradation	36	3	1.7E-1	4	1.4E-1	1	2.0E-1	1	2.8E-1
Sucrose degradation III	35	**6**	5.6E-3	**6**	2.2E-2	1	1.9E-1	1	2.9E-1
Xylose degradation	5	1	2.1E-1	**2**	3.9E-2	**1**	3.3E-2	1	2.1E-1

Grain yield-involved, genes involved in grain yield; GDMC-interaction, the grain yield-involved genes which negatively interacted with grain dry matter content; GDMC-involved, genes involved in grain dry matter content; n, number of genes; *p*, *p*-value for statistical significance. The symbol "-" represents data unavailable. The data in boldface represent significance at *p *< 0.05.

## Discussion

### Maize transcriptome at seedling stage

Gene expression of the parental inbred lines was profiled at the seedling stage. This strategy largely reduced the variance during plant collection, since seedlings can be grown in large quantities under highly controlled conditions [9]. Maize seedling transcriptome employed in our study did not take into account important trait-involved genes, which were regulated by developmental and environmental conditions. However, from previous research [5,6,10] it is known that grain yield associated genes (Table 1) were also regulated in ear or kernel development or stress response. This supports the hypothesis that the relative expression patterns of grain yield associated genes have already been established in early development stages [11]. Therefore the latent efficiency of these genes as determined at the seedling stage is expected to have a direct influence on grain yield.

### Two-step selection of trait-involved genes

Our newly developed two-step correlation approach targets at identifying all genes associated with grain yield and grain dry matter content using our expression and field data. On the one hand, it detects the most relevant genes in Step 1 using the stringent significance level of *p *< 0.0001. On the other hand, it also includes further important genes with the less stringent significance level of *p *< 0.01 on the basis of co-expression (*r *> 0.9). Employing co-expression reduced the number of about 2500 genes, which were significant at the 0.01 level, to 640. In conclusion, the two-step approach allows a more focused detection of relevant genes with a possibly important biological significance than solely a low statistical significance level. In Step 1, only 39 genes associated with both traits were detected. This number would have been too small to examine the interaction between the pathways involved in both traits. However, the additional genes identified in Step 2 enabled us to decode major interaction networks of grain yield and grain dry matter content (Table 1).

### Plant metabolism - sucrose degradation and glycolysis

Hexose phosphates derived from sucrose degradation are used to meet the energy and substrate requirements for plant growth. The finding that sucrose degradation was overrepresented in grain yield-involved genes (Table 3) suggests its significant role in maize production. Three genes encoding three types of invertases (MZ00005490, vacuolar invertase; MZ00026683, cytosolic invertase; MZ00033179, cell wall invertase) and one gene encoding a hexokinase (MZ00042300) were found to be positively associated with grain yield (Figure 2 and Table 1). This implies that sucrose degradation is up-regulated in high yielding hybrids, resulting in an increased hexose phosphate pool during the seedling stage (Figure 2). These results coincide with the fact that the strong relationship between invertase activity and growth rate was largely explained by common chromosomal regions co-located with genes encoding invertase and other related enzymes [12].

A considerable number of grain yield associated genes were found to be involved in glycolysis, an integrated (whole) plant metabolism using hexose phosphates (Table 3). PFK (MZ00013816, adenosine kinase/phosphofructokinase) is the principle enzyme regulating the entry of metabolites into glycolysis [13] through conversion of fructose-6-phosphate to fructose-1,6-bisphosphate. Its encoding gene was positively correlated with grain yield, indicating the up-regulation of glycolysis in high yielding hybrids. This result is supported by the fact that genes encoding alpha and beta subunits of PFP (Pyrophosphate-fructose 6-phosphate 1-phosphotransferase; MZ00024213 and MZ00024012, respectively), involved in interconversion of fructose-6-phosphate and fructose-1,6-bisphosphate, were both positively correlated with grain yield. These findings suggest that glycolysis is involved in grain yield, and the up-regulation of glycolysis seems to be a downstream effect of sucrose degradation up-regulation. This results in an increase of hexose phosphate, supplying more energy and more substrates, which are necessary for a strong seedling development. This deduction is supported by the fact that hexoses as well as sucrose have been recognized as important signal molecules in source-sink regulation and balance [14].

The relationship between carbohydrate metabolism and phytohormone signaling is illustrated by the fact that cytokinins enhance the gene expression of cell wall invertase and hexose uptake carriers [15]. One gene encoding a beta-glucosidase (MZ00035426) providing active cytokinins [16], one gene encoding a beta-glucosidase aggregating factor (MZ00013608) and a direct downstream gene of cytokinin (MZ00031351) encoding A-type response regulator [17] were positively associated with grain yield (Table 1). This suggests that up-regulated carbohydrate metabolism could partially be the result of cytokinin signaling regulation.

### Plant growth - cell expansion and endocycle

**Figure 3:**
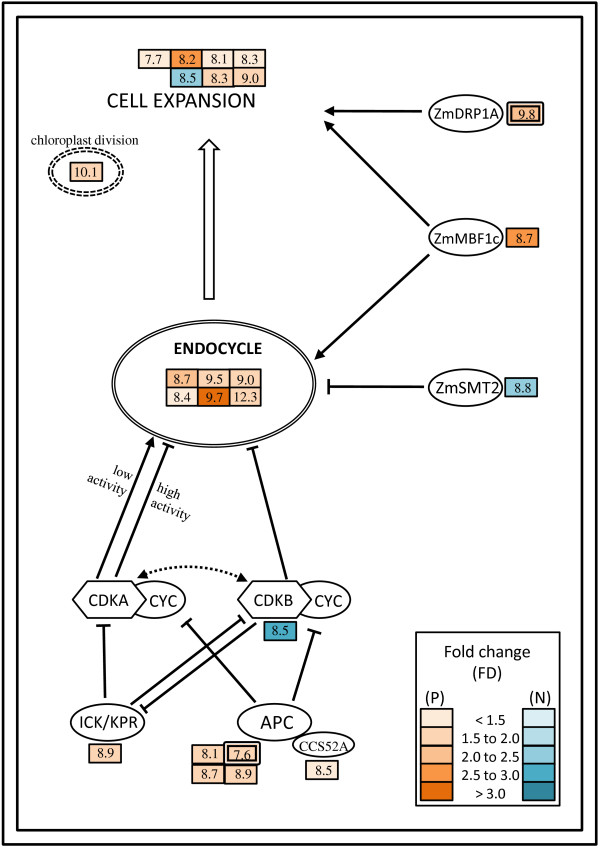
**Schematic representation of grain yield-involved genes in cell expansion and endocycle processes**. The rectangular boxes with the colored scales show the fold-changes (FD) of mid-parent expression for each gene. The mean mid-parent expression (log2 scale) is represented by the numbers in the boxes. Positively (P) and negatively (N) associated genes are shown in brown and blue, respectively. The boxes with two frames show genes with interactions to grain dry matter content (GDMC). The representation of the cell cycle genes regulating endocycle were taken from a previous review [25].

The growth of plant tissue generally proceeds in two stages. The first stage is cell division followed by cell expansion until differentiation is completed [18]. In an early developmental phase during endosperm development, cell division takes place and then organelle proliferation and cell expansion occur. In a later developmental phase, starch and proteins are deposited into the endosperm tissue. The early developmental phase decides over the final volume of the grain filling and consequently partly over the amount of final grain yield, due to the total cell number and the size of the cells [19]. In our results, the marker genes of cell expansion encoding V-type H^+^ATPase (MZ00013961) and aquaporins (MZ00043527) for water up-take [20] together with expansins (e.g. MZ00022872) and endo-1,3-beta-D-glucosidase (MZ00004156) for cell wall loosening [21], were positively associated with grain yield (Figure 3 and Table 1). This indicates that probably a high cell expansion rate in the seedling stage and maybe also later in the early phase of endosperm development is associated with high grain yield in hybrids. Larger cells, due to an increased cell expansion, have also been observed in maize roots of hybrids compared to their parental inbred lines [22]. The high expression of a gene (MZ00027266) encoding an FtsZ-like protein, which stimulates chloroplast division [23], indicates that hybrids with high grain yield may proliferate more chloroplasts along with cell expansion during seedling development and possibly also during endosperm development. This coincided with the regulation of genes located in the calvin cycle and chlorophyllide *a *biosynthesis (Table 3).

DNA synthesis, persisting after transition to cell expansion without subsequent cell division (M-phase), leads to endocycle, which significantly contributes to cell expansion in higher plants ([24] for review). The finding that the functional category CELL CYCLE AND DNA PROCESSING was overrepresented in grain yield associated genes (Table 2) suggests that this set of genes may play a significant role in grain yield regulation through their influence on endocycle, because most cells used for transcription profiling had already completed the cell division stage. For example, a gene (MZ00041750) encoding a DNA replication licensing factor and a gene (MZ00027598) encoding a subunit of a replication factor were positively associated with grain yield, which suggests that changes in the replication rate lead to alterations in the cell cycle of the hybrids. This deduction is also supported by the fact that several genes encoding enzymes involved in DNA repair were positively associated with grain yield. The ploidy level affects the cell size by increasing the metabolic output [25]. This supports the hypothesis that up-regulation of sucrose degradation and glycolysis in high yielding hybrids could be the result of a high ploidy level during cell expansion.

The endocycle is mediated by a down-regulation of cyclin-dependent kinase (CDK) activity in cells [25]. A gene (MZ00017440) encoding a B-type cyclin-dependent kinase (CDBK) was negatively associated with grain yield, implying that down-regulation of this *CDKB *could affect endocycle. Such a down-regulation could also be realized through less phosphorylation of CDK-inhibitors (ICK/KPRs) by CDKBs [26]. Another gene (MZ00021442) encoding ICK/KPR was also positively associated with grain yield, which stimulates the endocycle by decreasing the CDK activity. The activation of the ubiquitin-proteasome pathway [25] is a further mechanism to decrease CDK activity. The genes (e.g. MZ00020431) encoding the anaphase-promoting complex (APC) and another gene (MZ00030283) which encodes an APC-activating protein and belongs to the CCS52A class [27], were positively associated with grain yield. This suggests that the APC-dependent proteasome pathway may influence the endocycle through the proteolysis of cyclins and regulation of cyclin/CDK complexes. This deduction is consistent with previous results, where higher expression levels of *CCS52A *coincided with higher levels of endocycle in Medicago nodules [27].

Cell expansion and endocycle are also controlled by further mechanisms. The orthologue of *ZmDRP1A *(MZ00014057) is a positive factor for cell expansion in *Arabidopsis *[28,29]. In our study, it was positively associated with grain yield. In contrast, the orthologue of *ZmSMT2 *(MZ00056596) in *Arabidopsis *impedes endocycle [30]. In our study it was negatively associated with grain yield. This suggests the regulatory role of both genes in cell expansion during the maize seedling stage. Recently, a study demonstrated that transcriptional co-activators (*AtMBF1s*) play a significant role in controlling leaf cell expansion and the ploidy level [31]. From our results, a gene (MZ00003819; *ZmMBF1c*) encoding an orthologue of *AtMBF1c *was highly positively associated with grain yield and had a high fold-change across hybrids. This suggests that *ZmMBF1c *could significantly contribute to grain yield by controlling cell expansion along with regulating endocycle in the maize seedling.

Auxin is a phytohormone that regulates cell expansion and has been studied the most among all phytohormones [32]. Four genes (MZ00038300, MZ00021497, MZ00024781 and MZ00044325) encoding auxin-responsive factors were associated with grain yield, and also two genes (MZ00040986 and MZ00026772) encoding proteins for IAA modification. Furthermore, two genes possibly involved in IAA synthesis were associated with grain yield, indicating that the auxin signaling pathway could directly contribute to grain yield of maize hybrids throughout cell expansion.

### Overlap of pathways involved in grain yield and grain drymatter content

The fact that some metabolic genes were positively associated with grain yield but negatively associated with grain dry matter content suggests that overlaps exist at the metabolic level. A part of the grain yield associated genes located on regulatory or signaling pathways, such as the ubiquitin pathway or phytohormone pathways (Table 1 and Figure 3), were also associated with grain dry matter content, suggesting that regulatory genes involved in both traits are overlapping. When higher grain yield is achieved in breeding programs by accumulating genes positively associated with grain yield, these overlaps could lead to a decrease in grain dry matter content, resulting in higher post-harvest production costs due to artificial grain drying [3]. The selection of lines with a high expression of genes positively associated with one trait but at the same time not negatively with the second trait could result in a simultaneous increase of grain yield and grain dry matter content.

## Conclusions

We found that a high expression of genes involved in cell expansion, assessed at the parental lines of hybrids, was positively correlated with high grain yield of the hybrids. Therefore we hypothesize that hybrids with a high cell expansion rate have an advantage in growth and in grain development. At the same time, they probably can also provide more energy and substrates for growth, along with cell expansion. However, due to a negative correlation between grain yield and grain dry matter content, this latent ability of high yielding hybrids has a negative effect on grain dry matter content after harvest. Our study greatly extended the understanding of the mechanisms underlying grain yield at the molecular level. The results suggest that selection of inbred lines after transcript profiling at the seedling stage can help increase selection efficiency in maize breeding.

## Methods

### Field data

Seven flint and 14 dent elite inbreds developed in the maize breeding program of the University of Hohenheim were used as parental inbreds for 98 = 7 × 14 factorial crosses between both groups of inbreds. The inbreds comprised of eight dent lines with Iowa Stiff Stalk Synthetic background (S028, S036, S044, S046, S049, S050, S058, S067) and six with Iodent background (P033, P040, P046, P048, P063, P066). Four flint lines (F037, F039, F043, F047) had a European Flint background and three (L024, L035, L043) a Flint/Lancaster background.

The factorial crosses were evaluated in 2002 at six agroecologically diverse locations in Germany (Bad Krozingen, Eckartsweier, Hohenheim, Landau, Sünching, Vechta). The 21 inbred parents were evaluated for their *per se *performance in 2003 at four locations (Eckartsweier, Hohenheim, Sünching, Pocking) and in 2004 at three locations (Eckartsweier, Hohenheim, Bad Krozingen). The trials were evaluated in two-row plots using adjacent α designs with two to three replications. Hybrid performance for grain yield (*PY*) was assessed in Mg ha^-1 ^adjusted to 155 g kg^-1 ^grain moisture and hybrid performance for grain dry matter content (*PD*) in percent. The mid-parent heterosis of the hybrids for grain yield (*HY*) and grain dry matter content (*HD*) was determined. The field data were analyzed with a mixed linear model, which was described in detail in a previous study [33], where it was referred to as Experiment 1. The correlation between *PY *and *PD *was tested using a permutation test [34]. The distribution of the test statistic was approximated with Monte Carlo sampling using 9,999 samples.

### Microarray data

Seedlings of the 21 maize inbred lines were grown in a climate chamber under regulated growth conditions. RNA was isolated from a mixture of five seedlings of each line, which were 7 days old. The 46 k array from the maize oligonucleotide array project http://www.maizearray.org/, University of Arizona, USA) was used for transcription profiling [7]. For the microarray experiment an interwoven loop design [35] was applied. It resulted in 63 hybridizations of dent and flint lines by sampling each dent line five times and each flint line eight times. Blank and negative controls, which were located in all blocks of the array, were used to confirm the stability of the experiment. Because no *Spike-in *RNA was mixed into the isolated RNA, all *Spike-in *probes, were used as blank or negative controls. For experimental validation of the microarray experiment, two genes in eight different lines were evaluated by Quantitative RT-PCR, essentially in accordance with the microarray data. The microarray data were deposited in Gene Expression Omnibus (GEO) under the series accession GSE17754.

The gene-oriented probes with intensities (on a log2 scale) greater than the average intensity plus three times the standard deviation of all *Spike-in *probes were considered to be reliably expressed. Genes were further analyzed for differential expression, if their expression fold-changes between at least one pair of parental lines were greater than 1.3. The gene-oriented probes together with *Spike-in *probes were tested for statistically significant differential expression across all comparisons with a moderated *F*-test and subsequently with a nested *F*-test for each comparison of parental lines. The *LIMMA *package [36] was applied for the tests. According to the most significant *Spike-in *probe with an adjusted *p*-value of 0.049, a false discovery rate (FDR) of 0.01 was chosen as a more conservative cutoff in order to detect significant differential expression between inbred lines. For each differentially expressed gene, we calculated the average *L *of the expression level (log2 scale) in the parents of each hybrid.

### Correlation analysis

The correlations *r*(*L*, *PY*), *r*(*L*, *PD*) *r*(*L*, *HY*), and *r*(*L*, *HD*) between the average expression level of a gene in the parental lines and the hybrid performance and heterosis for grain yield and grain dry matter content, respectively, were determined. Significance of the correlations was tested with a *t*-test with *n *- 2 degrees of freedom, where *n *= 98 is the number of hybrids in the factorial. A type I error rate of 0.01 adjusted for multiple testing using a false discovery rate [37] was employed and the *p*-value of each gene was adjusted accordingly. Confidence intervals for the correlations were determined based on *Bca *(bias-corrected accelerated) bootstrap (α = 95%, 10,000 resamples) [38].

We employed a newly developed two-step correlation approach to identify genes associated with grain yield (Figure 1). In Step 1, all genes for which the correlations *r*(*L*, *PY*) or *r*(*L*, *HY*) were highly significant (*p *< 0.0001) were assigned to the set *S*. In Step 2, such genes that were not included in set *S *in the previous step but were highly correlated (*r *> 0.9) with genes included in set *S *in the previous step, were then added to *S*. Step 2 was iteratively repeated until no new genes were added to set *S*.

To determine a set of genes *T *associated with grain dry matter content we carried out a similar approach, but here only the correlations for hybrid performance *r*(*L*, *PD*) were considered in Step 1, because heterosis for grain dry matter content is low in maize [39].

The stability of the correlations was investigated with a cross validation procedure. In the cross validation, five dent and three flint lines were selected from the 7 × 14 factorial to compile the estimation set [40]. The set of trait associated genes was determined in the estimation sets generated by 100 rounds of cross validation. For each gene, it was determined how often it was assigned to the set of the trait associated genes in the 100 estimation sets. The genes were arranged according to this frequency and the sequence of the first 200 genes was compared to the sequence of the 200 genes with the smallest *p*-value determined from the complete data set. The difference between these two sets of genes was used as a measure for the instability of the correlations which were introduced by the genetic background.

### Pathway annotation

Comprehensive pathway annotation is the first step in mining the pathways underlying biological processes. The representative consensus sequences of all gene-oriented probes were searched using BLAST against the TIGR rice protein database http://www.tigr.org/, the TAIR *Arabidopsis *protein database http://www.arabidopsis.org/, and the Uniprot Knowledgebase http://www.ebi.ac.uk/, which includes the Swissprot Knowledgebase and the Trembl database. The functional annotations were assigned based on sequence similarity (*e*-value < 1e-5) with manual adjustment when necessary. Transcription factors, one of the most important components of regulatory networks, were organized into different gene families or sub-families based on the classification of the most similar rice transcription factors http://ricetfdb.bio.uni-potsdam.de/. Applying the same approach, protein kinases, located in signaling transduction pathways, were classified through the rice protein kinase database http://rkd.ucdavis.edu/. Genes involved in phytohormone signaling pathways were annotated by searching curated annotations (keyword item) of similar proteins in the Swissprot Knowledgebase. Cell cycle genes were re-annotated following the classification in *Arabidopsis *[41]. All gene-oriented probes were grouped into functional categories based on the MIPS Functional Catalogue of *Arabidopsis*, which is efficient for grouping cereal genes http://mips.gsf.de[42], and metabolic pathways based on RiceCyc http://www.gramene.org/pathway/. We identified the statistically enriched MIPS category or metabolic pathway of the trait-involved genes based on a background distribution employing the hypergeometric distribution [43].

## Abbreviations

*HD*: mid-parent heterosis for grain dry matter content; *HY*: mid-parent heterosis for grain yield; *PD*: hybrid performance for grain dry matter content; *PY*: hybrid performance for grain yield; *r*: correlation coefficient.

## Authors' contributions

JF conducted the statistical analysis, interpreted the results and wrote the paper; AT grew the plants, performed all microarray hybridizations and helped to write the paper; TAS gathered and analyzed the field data; AEM, SS, and MF devised and planned the study, contributed to the lab analysis, and contributed to the writing of the paper. All authors read and approved the final manuscript.

## Supplementary Material

Additional file 1 **Number of genes, which were differentially expressed in the parents of each hybrid of the factorial mating scheme**. A moderated *F*-test with a significance level of 0.01 and a fold change of at least 1.3 was used to detect the differentially expressed genes. Click here for file

Additional file 2 **List of trait-involved genes including comprehensive annotation**. The genes involved in grain yield and grain dry matter content (GDMC) were collected through Step 1 (F) and Step 2 (S). For each gene, the mean and the fold-change (FD) of mid-parent expression were calculated; positive (P) or negative (N) association to grain yield and GDMC is also provided. The correlation (*r*) of each gene with hybrid performance for grain yield (*PY*), mid-parent heterosis for grain yield (*HY*), hybrid performance for GDMC (*PD*) and the respective *p*-values (*p*) were listed. Click here for file
