# Supplementary material for: Dissecting grain yield pathways and their interactions with grain dry matter content by a two-step correlation approach with maize seedling transcriptome
Source: BMC Plant Biol. 2010 Apr 12;10:63. doi: 10.1186/1471-2229-10-63 (PMC2923537; doi:10.1186/1471-2229-10-63)
Supplement: Additional file 1 — Number of genes, which were differentially expressed in the parents of each hybrid of the factorial mating scheme. A moderated F-test with a significance level of 0.01 and a fold change of at least 1.3 was used to detect the differentially expressed genes. [file 1471-2229-10-63-S1.DOC]

**Additional file 1 - Number of genes, which were differentially expressed in the parents of each hybrid of the factorial mating scheme.**

| **Inbred** | **Gene** | **F037** | **F039** | **F043** | **F047** | **L024** | **L035** | **L043** |
| --- | --- | --- | --- | --- | --- | --- | --- | --- |
| **P033** | *number* | 3783 | 3339 | 3158 | 3620 | 1674 | 4233 | 3392 |
|  | *percentage* | 8.7% | 7.7% | 7.3% | 8.3% | 3.9% | 9.8% | 7.8% |
| **P040** | *number* | 3256 | 3149 | 3079 | 3505 | 2899 | 3522 | 3196 |
|  | *percentage* | 7.5% | 7.3% | 7.1% | 8.1% | 6.7% | 8.1% | 7.4% |
| **P046** | *number* | 3346 | 3892 | 3120 | 3873 | 2293 | 3462 | 3468 |
|  | *percentage* | 7.7% | 9.0% | 7.2% | 8.9% | 5.3% | 8.0% | 8.0% |
| **P048** | *number* | 3723 | 3984 | 4246 | 5159 | 3020 | 4293 | 3916 |
|  | *percentage* | 8.6% | 9.2% | 9.8% | 11.9% | 7.0% | 9.9% | 9.0% |
| **P063** | *number* | 3307 | 3545 | 3175 | 3812 | 2242 | 3392 | 4074 |
|  | *percentage* | 7.6% | 8.2% | 7.3% | 8.8% | 5.2% | 7.8% | 9.4% |
| **P066** | *number* | 2960 | 3165 | 2910 | 3876 | 1951 | 3514 | 3597 |
|  | *percentage* | 6.8% | 7.3% | 6.7% | 8.9% | 4.5% | 8.1% | 8.3% |
| **S028** | *number* | 3252 | 3262 | 3145 | 3811 | 2634 | 3169 | 3028 |
|  | *percentage* | 7.5% | 7.5% | 7.2% | 8.8% | 6.1% | 7.3% | 7.0% |
| **S036** | *number* | 3575 | 3442 | 3153 | 3832 | 3194 | 3660 | 3560 |
|  | *percentage* | 8.2% | 7.9% | 7.3% | 8.8% | 7.4% | 8.4% | 8.2% |
| **S044** | *number* | 2690 | 3411 | 2951 | 3888 | 2389 | 2988 | 2883 |
|  | *percentage* | 6.2% | 7.9% | 6.8% | 9.0% | 5.5% | 6.9% | 6.6% |
| **S046** | *number* | 2352 | 2681 | 2008 | 2782 | 1907 | 2661 | 2391 |
|  | *percentage* | 5.4% | 6.2% | 4.6% | 6.4% | 4.4% | 6.1% | 5.5% |
| **S049** | *number* | 2869 | 3012 | 2841 | 3342 | 2730 | 3404 | 2970 |
|  | *percentage* | 6.6% | 6.9% | 6.5% | 7.7% | 6.3% | 7.8% | 6.8% |
| **S050** | *number* | 2898 | 3315 | 2829 | 3316 | 2288 | 2836 | 3101 |
|  | *percentage* | 6.7% | 7.6% | 6.5% | 7.6% | 5.3% | 6.5% | 7.1% |
| **S058** | *number* | 3882 | 3878 | 3639 | 4597 | 3841 | 4413 | 4191 |
|  | *percentage* | 8.9% | 8.9% | 8.4% | 10.6% | 8.9% | 10.2% | 9.7% |
| **S067** | *number* | 4375 | 4415 | 4510 | 4793 | 3812 | 4306 | 4074 |
|  | *percentage* | 10.1% | 10.2% | 10.4% | 11.0% | 8.8% | 9.9% | 9.4% |

A moderated *F*-test with a significance level of 0.01 and a fold change of at least 1.3 was used to detect the differentially expressed genes.
